# Cilostazol for the prevention of pneumonia: a systematic review

**DOI:** 10.1186/s41479-018-0046-5

**Published:** 2018-04-05

**Authors:** Hirotaka Nakashima, Kazuhisa Watanabe, Hiroyuki Umegaki, Yusuke Suzuki, Masafumi Kuzuya

**Affiliations:** 1 Centre for Community Liaison and Patient Consultations, Nagoya University Hospital, 65 Tsurumai-cho, Showa-ku, Nagoya, Aichi 466-8560 Japan; 2 Department of Geriatrics, Nagoya University Hospital, Nagoya, Japan; 3 Department of Community Healthcare and Geriatrics, Nagoya University Graduate School of Medicine, Nagoya, Japan; 4 Institute of Innovation for Future Society, Nagoya University, Nagoya, Japan

**Keywords:** Antiplatelet, Cilostazol, Elderly, Pneumonia, Swallowing function

## Abstract

**Background:**

Pneumonia is a very common disease, especially among the elderly. Various drugs’ preventive effects against pneumonia have been reported. The antiplatelet drug cilostazol is used to prevent pneumonia, but the robustness of its efficacy is unclear. This review estimates the effectiveness of cilostazol for preventing pneumonia in elderly individuals.

**Methods:**

The following databases were searched from the earliest record to January 2016, without language restriction (the secondary search was conducted on February 2017): MEDLINE, Cochrane Library, CINAHL, and Ichushi-Web. Studies were included if they were published randomized controlled trials investigating the preventive effect of cilostazol on pneumonia in the elderly. The outcome was the incidence of pneumonia.

**Results:**

Two trials were identified that met the search criteria (1423 participants). Both trials compared cilostazol with no antiplatelet in patients with a history of cerebral infarction. A meta-analysis was not performed because of the small number of trials and the heterogeneity of the data. Both trials suggested that cilostazol reduced the incidence of pneumonia (risk ratio [RR] 0.40; 95% confidence interval [CI] 0.22–0.73 in one trial, RR 0.20; 95% CI 0.06–0.69 in the other) and the recurrence of cerebral infarction (0.43; 0.21–0.90, 0.53; 0.34–0.81, respectively). The quality of evidence provided by the trials was very low, mainly because of the high risk of bias.

**Conclusions:**

It is difficult to draw conclusions on the basis of two trials. Moreover, in the two trials, cilostazol could have reduced the incidence of pneumonia via a reduction of the recurrence of cerebral infarction, which suggests that other antiplatelets could also have the same effects. Stronger evidence is required from large trials assessing the effectiveness of cilostazol for the prevention of pneumonia.

**Trial registration:**

PROSPERO (CRD42016036724).

**Electronic supplementary material:**

The online version of this article (10.1186/s41479-018-0046-5) contains supplementary material, which is available to authorized users.

## Background

Pneumonia is a very common disease, especially in the elderly. The mortality rate of pneumonia has not decreased despite advances in antimicrobial therapy [1]. In Japan, the country with the most aged population in the world, pneumonia has risen from the fourth most common cause of death to the third most common [2]. A preventive effect of various drugs against pneumonia has been reported [3, 4] and cilostazol is one of these drugs.

Cilostazol is an antiplatelet agent that has been commercially available for more than two decades. It has antiplatelet and vasodilatory effects via the inhibition of phosphodiesterase-3 (PDE3), and it presents a relatively small risk of bleeding compared to other antiplatelet agents [5]. Many studies have demonstrated the effectiveness of cilostazol in the management of intermittent claudication due to peripheral arterial disease, cerebrovascular disease, and coronary artery disease [5]. Despite the benefit of cilostazol, some patients discontinue the drug due to adverse effects such as headache and increased heart rate [5], and cilostazol is contraindicated in patients with heart failure because of the risk for exacerbation.

Pneumonia in the elderly is often caused by silent aspiration resulting from reduced swallowing and coughing reflexes [6]. Substance P, a neurotransmitter in the nucleus of the solitary tract in the brainstem, plays important roles in these reflexes [7]. Cilostazol increases the level of substance P in the brain by inhibiting PDE3 [8] and thus could prevent pneumonia [9].

There are two systematic reviews that investigated the preventive effect of cilostazol on pneumonia, and the authors of those reviews concluded that although cilostazol seemed to help prevent pneumonia, the use of cilostazol was not recommended because of the risk of bleeding [3, 4]. However, those reviews did not focus on cilostazol; rather, the comprehensive prevention of pneumonia was the focus. Moreover, since those reviews were published, one randomized controlled trial (RCT) in this field has also been published [10].

In 2015, cilostazol was rated to have the best risk–benefit profile for secondary prevention after stroke among long-term antiplatelet mono- and dual therapies [11]. The frequency of cilostazol prescription is expected to increase in the future, and it would therefore be useful to elucidate its characteristics. This study represents an updated and specialized systematic review of RCTs to estimate the effectiveness of cilostazol in preventing pneumonia.

## Methods

The protocol of the present review is registered at PROSPERO (the International Prospective Register of Systematic Reviews, of the UK’s National Institute for Health Research) (#CRD42016036724) [12]. The methods used were based on the Cochrane Collaboration Handbook [13]. The recommendations of the Preferred Reporting Items for Systematic Reviews and Meta-Analyses (PRISMA) were followed [14].

### Search strategy

This review searched the following databases from the earliest record to 5 January 2016 without language restriction: MEDLINE (EBSCOhost), Cochrane Library, CINAHL (the Cumulative Index to Nursing and Allied Health Literature), and Ichushi-Web (Japan Medical Abstracts Society, http://www.jamas.or.jp). Keywords and MeSH terms relevant to the intervention of interest were used, including “cilostazol” and “antiplatelet,” and terms relevant to the medical condition of interest, including “pneumonia,” “aspiration,” and “dysphagia.” An additional file shows the full search strategies [see Additional file 1]. A manual search of relevant journals and reference lists of eligible studies was also carried out. The secondary search was conducted on 26 February 2017.

### Inclusion criteria

Studies were regarded as eligible for inclusion if they were published RCTs investigating the preventive effect of cilostazol on pneumonia in the elderly (i.e. patients ≥65 years old). Studies were included if they investigated the incidence of pneumonia, but other studies (such as studies on the swallowing function) were excluded. Studies of patients from community settings, long-term care settings, and hospitals were included. Studies of critically ill or ventilated patients were excluded. No restrictions were set on past medical history, concurrent medication (including antiplatelet medications), or the dosage of cilostazol. Eligible studies’ diagnostic criteria for pneumonia had to include symptoms or clinical signs. Studies were included if they followed up their subjects for > 1 month.

### Study selection

Two reviewers independently evaluated the studies for eligibility. Disagreements between the reviewers concerning the decision to include or exclude a study were resolved by consensus, and if necessary, by consultation with a third reviewer. After the titles and abstracts retrieved from the searches were assessed, the full texts of the studies were assessed for eligibility. Those studies fulfilling the eligibility criteria were included.

### Data extraction and quality assessment

Data were extracted by two independent reviewers, and disagreements were resolved by consensus. Extracted data included details on the study design, setting, participants, intervention, control therapy, diagnostic criteria, and incidence of pneumonia. Missing data were not imputed. The two reviewers also independently assessed the risk of bias for each study included by using the Cochrane Collaboration risk of bias tool [13]. It was planned that reporting bias would be assessed using the visual asymmetry of a funnel plot if there were at least 10 trials.

### Statistical analysis

The primary outcome was the incidence of pneumonia. A random-effects meta-analysis with risk ratios (RR) for binary outcomes was performed, and 95% confidence intervals (CI) and two-sided *p*-values using the software Review Manager 5.3 (RevMan, [Computer program] Version 5.3. Copenhagen: The Nordic Cochrane Centre, The Cochrane Collaboration 2014) were calculated. The review assessed heterogeneity between the studies in terms of their effect measured by the visual inspection of forest plots and the I^2^ statistic. A subgroup analysis regarding the dosage of cilostazol was performed, and the subsequent sensitivity analyses included only (1) trials with a low risk of bias, and (2) trials comparing cilostazol with other antiplatelet medications. The Grades of Recommendation, Assessment, Development and Evaluation (GRADE) approach was used to assess the overall certainty of evidence [15].

## Results

### The search results

**Fig. 1:**
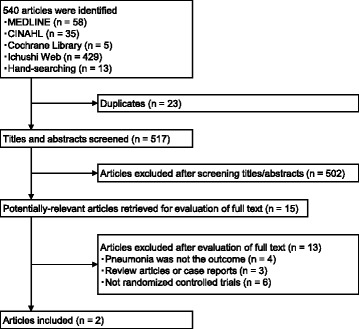
Flow of studies through the review

As illustrated in Fig. 1, search strategies identified 517 reports without duplicates. The screening of the titles and abstracts of these reports identified 15 reports as potentially eligible for this review. The full-text screening of these 15 reports excluded 13 reports. The remaining two RCTs met the inclusion criteria [10, 16].

### Included studies

The two trials, which are described in detail in Table 1, examined 1423 randomized patients: the Yamaya et al. trial included 328 patients and was published in 2001 [16], and the Shinohara et al. trial investigated 1095 patients and was published in 2006 [10]. Both trials compared cilostazol with the absence of antiplatelet medication in patients with a history of cerebral infarction in Japan. The control groups of these trials did not take aspirin, because the use of aspirin to prevent the recurrence of cerebral infarction was not approved by the Japanese Ministry of Health, Labor and Welfare at that time.

**Table 1:** Summary of the two included studies

Study and country	Yamaya et al. 2001 [16], Japan	Shinohara et al. 2006 [10], Japan
Population	History of cerebral infarction but not bedridden	History of cerebral infarction, age < 80 years
Mean age	Mean age: 76.5 ± 2.1 years	Mean age: 65.0 ± 8.7 years
Gender	No statement about gender	Female 34.4%
Risk factors	No statement about risk factors for pneumonia	No statement about risk factors for pneumonia
Criteria for diagnosis of pneumonia	New pulmonary infiltrate seen on chest radiographs (assessed by two radiologists not involved in the study) and one of the following: cough, body temperature > 37.8 °C, subjective dyspnea	Description by attending physician in the medical chart, chest radiographs and blood test
Intervention and control (n, allocated)	I: cilostazol 100 mg per day (n: not stated)	I: cilostazol 200 mg per day (n: 547)
	C: no active treatment (n: not stated)	C: placebo (n: 548)
	328 patients were allocated in total but the number of patients in each group at allocation was not reported	
Intervention period	3 years	3.3 years on average
Number of patients analyzed	*I* = 125, C = 145, see comments	*I* = 531, C = 533, see comments
Outcomes	Pneumonia	Pneumonia
	I: 12 (9.6%), C: 35 (24.1%)	I: 3 (0.56%), C: 15 (2.8%)
	RR = 0.40 (95% CI 0.22–0.73)**	RR = 0.20 (95% CI 0.06–0.69)**
	Cerebral infarction	Cerebral infarction [17]
	I: 9 (7.2%), C: 24 (16.6%)	I: 30 (5.6%), C: 57 (10.7%)
	RR = 0.43 (95% CI 0.21–0.90)*	RR = 0.53 (95% CI 0.34–0.81)*
	Bleeding	Bleeding [17]
	I: 12 (9.6%), severity of the bleeding events was not reported	Fatal intracranial hemorrhage
	C: No statement about adverse events	I: 0, C: 1
		Nonfatal intracranial hemorrhage
		I: 4 (0.75%), C: 6 (1.1%)
		Other bleeding
		I: 15^a^ (2.8%), C: 11 (2.1%)
Risk of bias		
Random sequence generation	Low	Low
Allocation concealment	Unclear: Inadequate description	Low
Blinding of participants and personnel	High: Open-label trial	Low
Blinding of outcome assessment	Low	Unclear: Post-hoc analysis of a RCT [17]
Incomplete outcome data	High: As-treated analysis	Unclear: Reasons for dropouts were unclear
Selective reporting	High: No protocol available, No statement about adverse events in the control group	High: Post-hoc analysis of a RCT [17]
Other bias	Unclear: No statement about funding	Unclear: No statement about funding but original RCT [17] was supported by Otsuka Pharmaceutical
Comments	Reported in a Letter to the Editor	1095 patients were randomized but 31 patients were excluded from analysis because of “serious protocol violations”
	328 patients were randomized but 58 patients were excluded from analysis; 31 patients died from causes other than pneumonia; additionally, 27 patients in cilostazol group dropped out because of adverse events	Of 1064 patients, about half of the patients discontinued the treatment (I: 54%, C: 49%)

*C* control, *CI* confidence interval, *I* intervention, *RR* risk ratio

*P < 0.05, **P < 0.01

^a^ Including one gastrointestinal bleeding

The study by Yamaya et al. was designed to establish whether cilostazol lowers the incidence of pneumonia and reduces the recurrence of cerebral infarction in patients with a history of stroke [16]. The trial compared cilostazol with no active treatment without placebo. Because the trial was only reported as a ‘Letter to the Editor’, relatively few data from the trial appeared in the published article.

The study by Shinohara et al. [10] was a post-hoc analysis of the Cilostazol Stroke Prevention Study (CSPS) [17]. The CSPS was a multicenter, randomized, placebo-controlled double-blind study, and the CSPS group reported the efficacy and safety of cilostazol for the secondary prevention of cerebral infarction.

### Risk of bias in the two trials

Both the Yamaya et al. and Shinohara et al. trials had varying risks of bias, as follows.

#### Allocation

Both trials reported using random sequence generation processes and had low risks of bias. Regarding allocation concealment, the study by Yamaya et al. had an unclear risk of bias because the study’s authors reported only that the allocation list was held independently of the investigators.

#### Blinding

In the Yamaya et al. study, blinding of the participants was impossible, and the diagnosis of pneumonia required symptoms that had to be assessed subjectively. This trial therefore had a high risk of bias. On the other hand, the trial had a low risk bias in terms of the blinding of the outcome assessment because radiologists who were not involved in the study diagnosed the participants’ pneumonia. The Shinohara et al. study was a post-hoc analysis of a double-blind trial. The study had an unclear risk of bias for outcome assessment because the outcome assessors might have had information on the interventions used.

#### Incomplete outcome data

Yamaya et al. performed an as-treated analysis, so the trial had a high risk of bias. The study by Shinohara et al. had an unclear risk of bias because the reasons for post-randomization dropouts were unclear.

#### Selective reporting

The study protocol of Yamaya et al. study was not able to be obtained. Those authors did not report adverse events in the control group (no active treatment), and the trial had a high risk of bias. The study by Shinohara et al. had a high risk of bias because the study was a post-hoc analysis of the CSPS [17] and it did not set pneumonia as an outcome.

#### Other potential sources of bias

Both studies did not report any funding, but the CSPS [17] was supported by Otsuka Pharmaceutical Co., Ltd.

### The effects of intervention

**Fig. 2:**
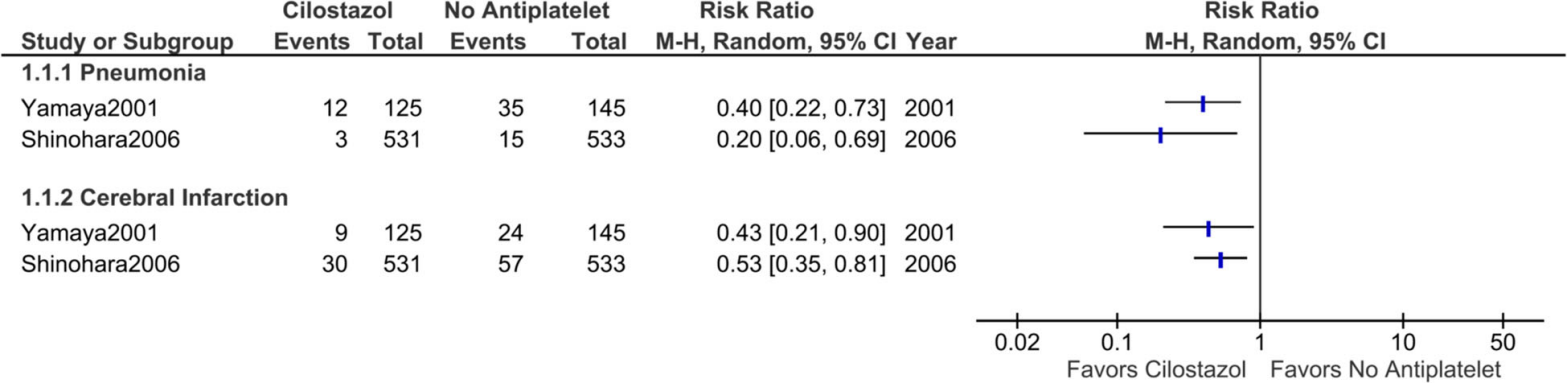
Risk of pneumonia and cerebral infarction with cilostazol treatment compared with no antiplatelet. A meta-analysis was not performed because of the small number of trials. Yamaya et al. (2001) [16] compared cilostazol with no active treatment without placebo. Shinohara et al. (2006) [10] compared cilostazol with placebo

A meta-analysis was not performed because of the small number of trials and the heterogeneity of data (the heterogeneity will be described later in detail). Hence, the outcomes were addressed using a narrative description of the available evidence. Both the Yamaya et al. and Shinohara et al. studies described that the cilostazol groups had significantly lower incidences of pneumonia compared to the groups that did not use antiplatelets (RR 0.40; 95% CI 0.22–0.73 in Yamaya et al., RR 0.20; 95% CI 0.06–0.69 in Shinohara et al.) (Fig. 2). The cilostazol groups also had lower risks for the recurrence of cerebral infarction (RR 0.43; 95% CI 0.21–0.90, RR 0.53; 95% CI 0.34–0.81, respectively).

The incidence of pneumonia was considerably higher in the Yamaya et al. study (17.4%) compared to the Shinohara et al. study (1.7%). This was largely because the patients in the Yamaya study were older than those in the Shinohara study. The age difference could also affect the recurrence of cerebral infarction (12.2% in the Yamaya study; 8.2% in the Shinohara study), as advanced age is one of the major risk factors of pneumonia.

### Adverse events

Yamaya et al. reported that 27 of the 152 (18%) patients who received cilostazol suffered adverse events: bleeding (12 patients), palpitation and/or tachycardia (11 patients), diarrhea (one patient), and headache (two patients). The severity of the bleeding was not reported. These 27 patients dropped out of the study. There was no statement about adverse events in the control group (no active treatment), suggesting that the trial did not collect or report the data on adverse events in the control group.

**Fig. 3:**
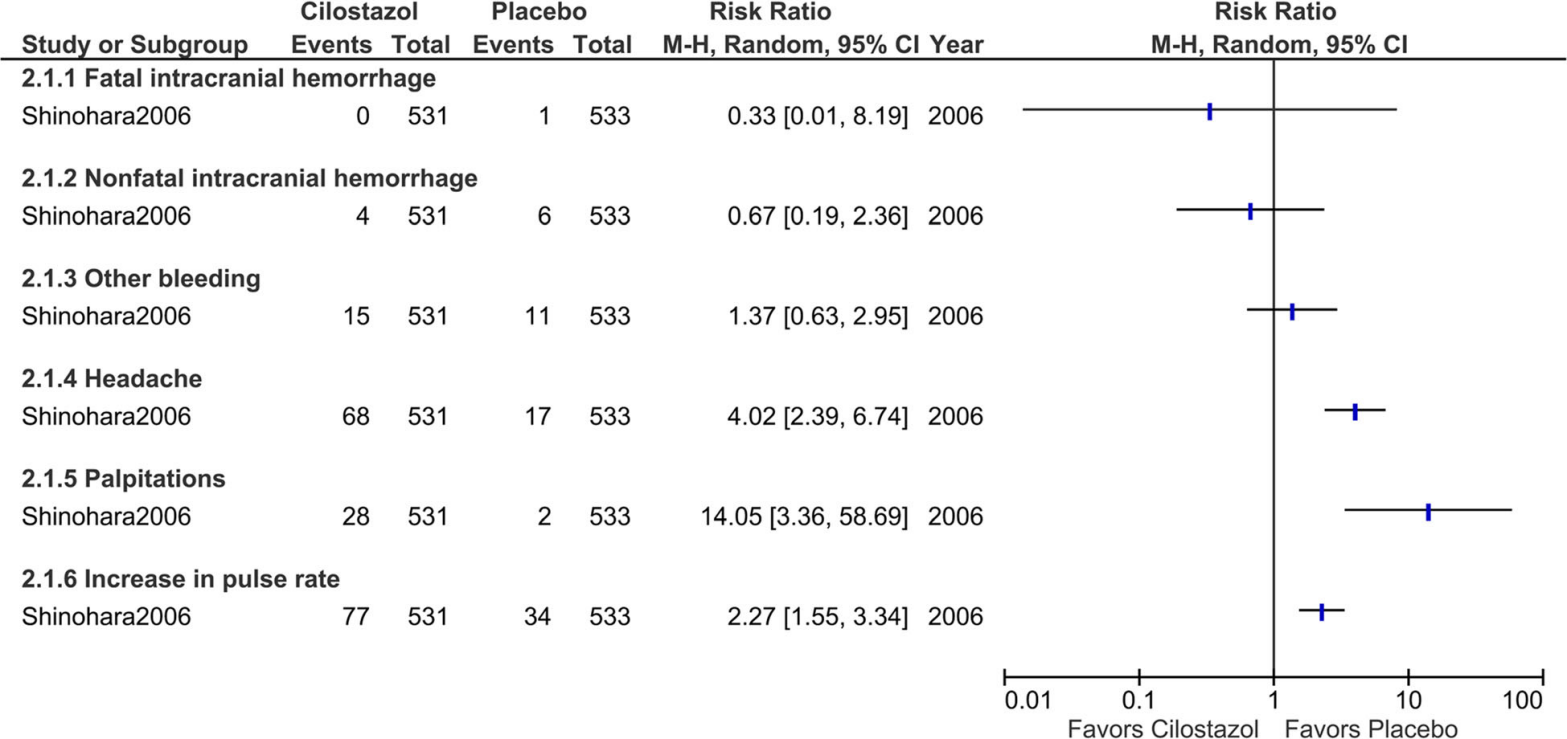
Risk of adverse events with cilostazol treatment compared with placebo. Only one trial (Shinohara et al. 2006) was included in this figure because the other (Yamaya et al. 2001) did not describe adverse events in the control group

The details of the adverse events in the Shinohara et al. study were provided in the CSPS report [17] (Fig. 3). The incidence of bleeding events was not significantly different between the cilostazol and placebo groups (2.8% and 2.1%, respectively). The bleeding events included one gastrointestinal bleeding event in the cilostazol group. Apart from those bleeding events, 11 patients developed intracranial hemorrhages; one patient in the placebo group developed a fatal intracranial hemorrhage; 4 patients in the cilostazol group and 6 in the placebo group developed nonfatal intracranial hemorrhages. Headache (12.8% in the cilostazol group and 3.2% in the placebo group), palpitations (5.3% and 0.4%, respectively), and an increase in pulse rate (19% and 7.9%, respectively) occurred with greater frequency in the cilostazol group, and these adverse events resulted in a higher discontinuation rate in the cilostazol group.

### Heterogeneity

Because only two trials were included in the present review, a formal testing of heterogeneity was not performed. However, the two trials were heterogeneous with respect to the following: the subjects’ ages, the dosage of cilostazol, the interventions in the control groups (no active treatment or placebo), the incidence of pneumonia, the discontinuation rate, and the methods of analysis (as-treated analysis or intention-to-treat analysis).

### Subgroup analyses, sensitivity analyses

A subgroup analysis or sensitivity analysis of the two trials was not performed.

### Reporting bias

Reporting bias using a funnel plot was not explored because of the small number of trials in this review.

### Excluded studies

Osawa et al. retrospectively compared cilostazol with the absence of cilostazol (most patients took some antiplatelets other than cilostazol) with regard to the preventive effect on pneumonia in patients with a history of cerebral infarction [18]. This study was excluded because it was not a RCT.

Teramoto et al. reported their double-blinded, placebo-controlled, three-period crossover study that compared the effects on the swallowing function of cilostazol, aspirin, and placebo in patients with a history of cerebral infarction [9]. This study was also excluded, because it did not examine the incidence of pneumonia.

## Discussion

Only two trials met the inclusion criteria for this study. Both trials compared cilostazol with the absence of any antiplatelet medication in patients with a history of cerebral infarction in Japan. Both trials showed that cilostazol significantly reduced the incidence of pneumonia and the recurrence of cerebral infarction.

According to the GRADE system [15] that we used to assess the quality of evidence, the two trials provide very low-quality evidence. The evidence was downgraded from high to very low quality because of a very high risk of bias (downgraded two levels) and imprecision due to the small sample size (one level).

Two systematic reviews investigated the preventive effect of cilostazol on pneumonia [3, 4], and both were published before the study by Shinohara et al. and therefore included only the Yamaya et al. study. The Shinohara et al. study, which was a larger placebo-controlled trial, was added to our systematic review.

The present review has a major limitation. Both of the trials examined herein compared cilostazol with the absence of antiplatelets, which resulted not only in a decrease in the incidence of pneumonia but also a low risk of the recurrence of cerebral infarction in the cilostazol group. Cerebral infarction is one of the major risk factors of pneumonia, and thus cilostazol could reduce the incidence of pneumonia by reducing the recurrence of cerebral infarction. This means that other antiplatelets could also have the same effect in terms of preventing pneumonia.

As described above, there is no conclusive evidence to recommend cilostazol to prevent pneumonia. However, Teramoto et al. reported that cilostazol but not aspirin improved the swallowing function of patients with a history of cerebral infarction [9]. In addition, Osawa et al. reported that in their retrospective study of patients with a history of cerebral infarction, the incidence of pneumonia was lower in the cilostazol group compared to the group in which most patients (95%) took an antiplatelet other than cilostazol [18]. These studies suggest that cilostazol but not other antiplatelet medications has a preventive effect on pneumonia by improving the swallowing function.

Further trials are needed to assess the preventive effects of cilostazol against pneumonia compared to other antiplatelets. Other trial designs would also be useful; for example, the co-administration of cilostazol and other antiplatelets. In the current clinical setting, cilostazol is used as an additional treatment to aspirin or clopidogrel, and thus such trial designs would provide more clinically meaningful outcomes. Trials comparing cilostazol with the absence of antiplatelets in patients who do not need to take antiplatelets would also be beneficial. Such trials could clarify the use of cilostazol as a preventive agent for pneumonia, rather than an antiplatelet. Cilostazol’s potential risk of bleeding complications is an issue, but dosage adjustment might resolve the problem.

## Conclusions

There is insufficient clinical trial evidence regarding the efficacy of cilostazol for preventing pneumonia. The result of two trials (Yamaya et al. and Shinohara et al.) suggested that cilostazol, compared to the absence of any antiplatelet medication, significantly reduced the incidence of pneumonia. However, these trials provided very low-quality evidence, mainly because of the high risk of bias. Moreover, in the two trials, cilostazol treatment may have reduced the incidence of pneumonia by reducing the recurrence of cerebral infarction, which suggests that other antiplatelets can also have the same effect. Stronger evidence is required from large trials assessing the effectiveness of cilostazol for the prevention of pneumonia.

## Additional file

Additional file 1 Full search strategies conducted on 5 January 2016. Description of data: Full search strategies on each database. (PDF 48 kb)

## Abbreviations

CI: Confidence interval
CSPS: Cilostazol Stroke Prevention Study
GRADE: Grades of Recommendation, Assessment, Development and Evaluation
RCT: Randomized controlled trial
RR: Risk ratio
