# Supplementary material for: Cilostazol for the prevention of pneumonia: a systematic review
Source: Pneumonia (Nathan). 2018 Apr 5;10:3. doi: 10.1186/s41479-018-0046-5 (PMC5885313; doi:10.1186/s41479-018-0046-5)
Supplement: Supplementary file 1 — Full search strategies conducted on 5 January 2016. Description of data: Full search strategies on each database. (PDF 48 kb) [file 41479_2018_46_MOESM1_ESM.pdf]

Additional file 1

Title of data: Full search strategies conducted on January 5th, 2016

Article title: Cilostazol for Prevention of Pneumonia: Systematic Review

Journal name: Pneumonia

Correspondence: Hirotaka Nakashima, E-mail: naka621@med.nagoya-u.ac.jp

MEDLINE (EBSCOhost)

1- “cilostazol”
2- (MH “Platelet Aggregation Inhibitors”)
3- (MH “Pneumonia+”)
4- (MH “Respiratory Aspiration+”)
5- (MH “Deglutition Disorders+”)
6- 1 OR 2
7- 3 OR 4 OR 5
8- 6 AND 7

Cochrane Library

1- “cilostazol”
2- Mesh [Platelet Aggregation Inhibitors] explode all trees
3- “antiplatelet agent*”
4- “antiplatelet drug*”
5- “antiplatelet*”
6- 1 or 2 or 3 or 4 or 5
7- Mesh [Pneumonia] explode all trees
8- Mesh [Respiratory Aspiration] explode all trees
9- Mesh [Deglutition Disorders] explode all trees
10- 7 or 8 or 9
11- 6 and 10

CINAHL

1- “cilostazol”
2- (MH “Platelet Aggregation Inhibitors+”)
3- “antiplatelet agent*”
4- “antiplatelet drug*”
5- “antiplatelet*”
6- 1 or 2 or 3 or 4 or 5
7- (MH “Pneumonia+”)
8- (MH “Aspiration”)
9- (MH “Deglutition Disorders”)
10- 7 OR 8 OR 9
11- 6 AND 10

Ichushi Web

1- Cilostazol/TH or “cilostazol”
2- Platelet Aggregation Inhibitors/TH or “antiplatelet”
3- Pneumonia/TH or “pneumonia”
4- Respiratory Aspiration/TH or “aspiration”
5- (1 or 2) and (3 or 4)
